# Pro-Inflammatory Markers in Relation to Cardiovascular Disease in HIV Infection. A Systematic Review

**DOI:** 10.1371/journal.pone.0147484

**Published:** 2016-01-25

**Authors:** Alinda G. Vos, Nikmah S. Idris, Roos E. Barth, Kerstin Klipstein-Grobusch, Diederick E. Grobbee

**Affiliations:** 1 Julius Center for Health Sciences and Primary Care, University Medical Center Utrecht, Utrecht, The Netherlands; 2 Department of Infectious Diseases, University Medical Center Utrecht, Utrecht, The Netherlands; Rutgers University, UNITED STATES

## Abstract

**Background:**

In the past years many inflammatory markers have been studied in association with clinically manifest cardiovascular disease (CVD) and carotid intima-media thickness (CIMT) in HIV-infected patients, to obtain insights in the increased cardiovascular risk observed in HIV infection. This systematic review provides an oversight of the current knowledge.

**Methods:**

A search was performed in PubMed, Embase and Cochrane in July 2014, identifying all articles from 1996 onwards addressing the relation between inflammatory markers and CVD or CIMT in HIV-positive adults. Two authors, using predefined criteria, independently conducted the selection of articles, critical appraisal and extraction of the data. Analysis was focused on the immune markers that were most frequently assessed. The review protocol was registered in the PROSPERO database at 11 July 2014 (registration number CRD42014010516). This review was performed according to the PRISMA guideline.

**Findings:**

Forty articles were selected; eight addressing cardiovascular disease (CVD) and thirty-two addressing CIMT. C-reactive protein (CRP), interleukin-6 (IL-6) and d-dimer were assessed most frequently in relation to the occurrence of CVD; in four out of eight studies. All three markers were positively related to CVD in three out of four studies. Studies addressing CIMT were too heterogeneous with respect to patient populations, inflammatory markers, CIMT measurement protocols and statistical methods to allow for a formal meta-analysis to obtain summary statistics. CRP, IL-6 and soluble vascular cell adhesion molecule (sVCAM-1) were the most studied markers in relation to CIMT. None of the inflammatory markers showed an association with CIMT.

**Interpretation:**

This review showed a relation between some inflammatory markers and CVD, however, no consistent relation is observed for CIMT. Statistical approaches that yields effect estimates and standardized CIMT protocols should be chosen. Further research should focus on prospective studies and a selected set of inflammatory markers.

## Introduction

When the human immunodeficiency virus (HIV) was discovered in the 1980’s, the infection was believed to be immunosuppressive. This view changed in the 1990’s, when evidence became available supporting the presence of chronic inflammation rather than primary immunodeficiency.[1]

With the initiation of antiretroviral therapy, mortality patterns in HIV patients changed from AIDS related opportunistic infections and malignancies to cancers not related to AIDS and cardiovascular disease (CVD).[2] Nearly ten years after the introduction of highly active antiretroviral therapy (HAART) non-AIDS defining illnesses were considered to be responsible for almost 50% of deaths in HIV-positive cohorts in North America; seven to 19% of all deaths were attributed to CVD.[3–5]

Chronic immune activation has a pivotal role in the pathogenesis of atherosclerosis in non-HIV infected patients.[6,7] Moreover, a range of studies has reported an association between immune activation and accelerated atherosclerosis in patients who are HIV-infected.[8–10]

The role of immune markers in relation to CVD risk in HIV-positive patients has not been clarified. Evaluating available data concerning the relation between pro-inflammatory parameters and CVD remains difficult if only because of differences in study design and the availability of various immune markers. Moreover, outcome measures vary from clinical relevant outcomes, like the occurrence of myocardial infarction or cardiac death, to surrogate markers of CVD: notably carotid intima-media thickness (CIMT) and markers of arterial stiffness.

The aim of the current review is to summarize the data on the association of pro-inflammatory markers with CVD, including their prognostic value, in HIV-infected patients.

## Methods

### Search strategy

The review protocol was registered in the PROSPERO database at 11 July 2014 (registration number CRD42014010516). A systematic literature search was conducted in PubMed, EMBASE and Cochrane library (Table 1). Words and synonyms related to the domain, determinant and outcome were used. The domain were HIV-infected adults. As determinant, plasma or serum immune markers were included. We excluded cellular blood components (i.e. lymphocyte subsets) and genetic markers. Symptomatical cardiovascular disease or surrogate markers for cardiovascular disease (i.e. CIMT, ankle brachial index) were considered as outcomes (S1 Table). Search terms were limited to title and abstract.

**Table 1:** Search strategy.

		Search terms	Pubmed (Medline) [title/abstract]	EMBASE [title/abstract]	Cochrane [title/abstract]
**#1 domain**	HIV positive patients	HIV			
		human immunodeficiency virus			
		human immuno deficiency virus			
		human immunedeficiency virus			
		human immune deficiency virus			
		aids			
		acquired immunodeficiency syndrome			
		acquired immuno deficiency syndrome			
		acquired immunedeficiency syndrome			
		acquired immune deficiency syndrome			
**AND**			309067	358649	16040
**#2 determinant**	Pro-inflammatory markers	Inflammatory			
		Inflammation			
		Inflamm*			
		Biomarker			
		Biomarkers			
		Immune*			
**AND**			985859	1262812	34151
**#3 Outcome**	Cardiovascular disease or surrogate markers of cardiovascular disease.	cardiovascular			
		CVD			
		Myocardial infarction			
		Mi			
		Coronary heart disease			
		CHD			
		Stroke			
		Carotid intima-media thickness			
		CIMT			
		Arterial stiffness			
		Flow mediated dilation			
		FMD			
		PWV			
		Pulse Wave Velocity			
		Coronoary artery calci*			
		CAC			
		Ankle brachial index			
		ABI			
			570481	476332	57183
**Final number of studies by combining #1 AND #2 AND #3**			**821**	**246**	**70**
*Search date for all databases July 2*, *2014*				*EMBASE (AND [embase]/lim NOT [medline]/lim)*	*1 cochrane review*, *68 trials*, *1 methods study*

Duplicates were removed by using reference management software, and further checked manually. The review was conducted in accordance to the PRISMA and STROME-ID guidelines.[11,12]

### Study selection

**Fig 1:**
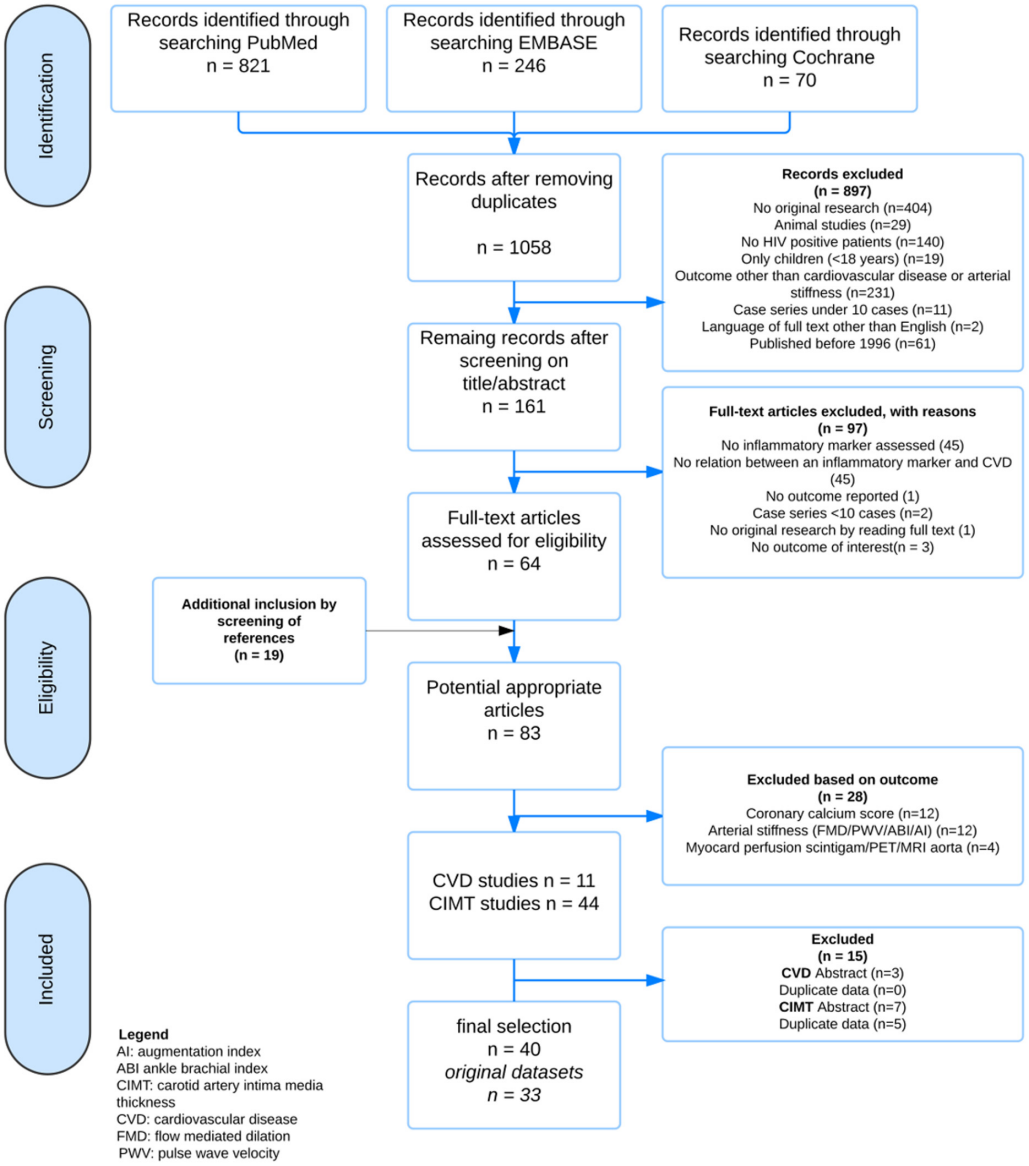
Flowchart inclusion. AI: augmentation index, ABI: ankle brachial index, CIMT: carotid intima media thickness, CVD: cardiovascular disease, FMD: flow mediated dilation, PWV: pulse wave velocity.

Study selection was done in three steps (Fig 1). First, all identified records were screened based on titles and abstracts by one author (AV). Second, full text reports of all abstracts were independently read to assess eligibility by two authors (AV, NI), using preset inclusion criteria. Third, references and citations of the selected articles were screened for additional articles. Discrepancies were discussed in a consensus meeting by two authors (AV, NI).

Agreement could be reached for all but one article as there were different opinions on the question whether there was a relation between the immune marker and outcome, or not. After consulting of a third reviewer (KK), the article was excluded. If the same group of patients was described in more than one article the most detailed report was included. If the reports were complementary both were included and data were combined. For studies describing a group of HIV-positive and HIV-negative individuals, only findings of HIV-positive participants were used.

### Validity and data extraction

The following data were extracted: year of publication, study design, follow-up duration, number of patients, country, setting, age, sex, years since HIV diagnosis, CD4 level, viral load, ART use and duration, classic cardiovascular risk factors, inflammatory parameters measured, outcomes and outcome measurement methods. ‘In case a database was described in more than one study, baseline characteristics of the most comprehensive article were used.’

Selected studies were critically appraised, particularly for the risk of selection-, detection-, and attrition bias. Bias risk was assigned as likely, unlikely, or unknown. The first author (AV) conducted the data extraction and critical appraisal using a set format. The second author (NI) independently checked all extracted data.

### Analysis

As studies were expected to be very heterogeneous, results are descriptive, grouped by outcome and, inflammatory marker. When possible, percentages of common baseline characteristics were calculated. Due to heterogeneity of the data it was impossible to present effect estimates in a clear overview. The only common estimate per study was a p-value; therefore p-values were presented in a figure, stratified by method of analysis and accompanied by the sample size. All p-values of 0.25 or higher were considered to express minimal association. When only ‘no significant’ was reported, the p-value in the figure was also set at 0.25, when a p-value of <0.05 was reported, a value of 0.03 was displayed in the figure. Outcome data did not allow calculations of summary statistics or prognostic value. A correlation was considered relevant if the Rho value was 0.4 or higher. Relevant correlations were depicted with a circle in the figure. The three most commonly studied inflammatory markers were analyzed separately. Besides the top-three-studied immune markers, findings of the remaining markers assessed at least thrice were summarized in a table. Differences in CIMT measurement protocols were not taken into account. In this review C-reactive protein (CRP) refers to both the regular CRP measurement as to the high-sensitive CRP assays.

## Results

1058 studies were identified, 64 articles remained after screening (Fig 1). Screening of references yielded another 19 articles, which did not mention immune marker measurement (mainly CRP) in title or abstract. Agreement for inclusion of articles by the two authors (AV, NI) was over 99%.

Due to incomplete information abstracts were excluded (CVD 3 abstracts, CIMT 7 abstracts), in deviation of the initial review protocol. Two studies addressing CVD used the SMART cohort data; both were included in the final analysis since they presented additional information.[9,13] Six populations studied for CIMT were described in more than one article.[14–29] Studies containing additional information remained in the final analysis,[17,19–25,29,30], studies presenting duplicate data were excluded.[14,16,27,28,31] Finally 40 articles remained (8 assessing CVD, 32 assessing CIMT) [9,10,13,15,17–25,29,30,32–56], including 33 original datasets, describing 48 immune markers.

### Baseline characteristics

Almost all studies addressing CVD had a case-control design (S1 Table). The number of cases ranged from 35 to 487 cases [51,56] The majority of patients were men, aged around 47 years. The most frequently assessed markers were CRP, IL-6 and d-dimer; all were assessed in five out of eight studies.

The vast majority of studies addressing CIMT were cross-sectional. Only six out of 32 CIMT studies had a prospective design. The average number of HIV-positive patients per study was 155 (median 129), 80% of which was male. The median age was 46 years and median duration since HIV diagnosis was 9.3 years (interquartile range (IQR) 6·3–13·0). 12 studies had ART coverage of 100%[18,33,37–40,43–45,48–50,57] and three datasets described only ART naïve patients.[19,20,36,47] Average ART coverage in the other studies was 71%, and ART duration was five years (mean and median). Twelve studies were performed in the USA, nine in Europe and one in Africa (Uganda). Nearly 45% of all HIV patients were current smokers and mean body-mass index was 25kg/m^2^. About 39% of studies specified that plasma was used, mostly frozen, for immune marker measurement. Protocols for CIMT measurements varied from two-point unilateral measurements to a comprehensive protocol with 12 measurements in each carotid artery.

### Critical appraisal

**Fig 2:**
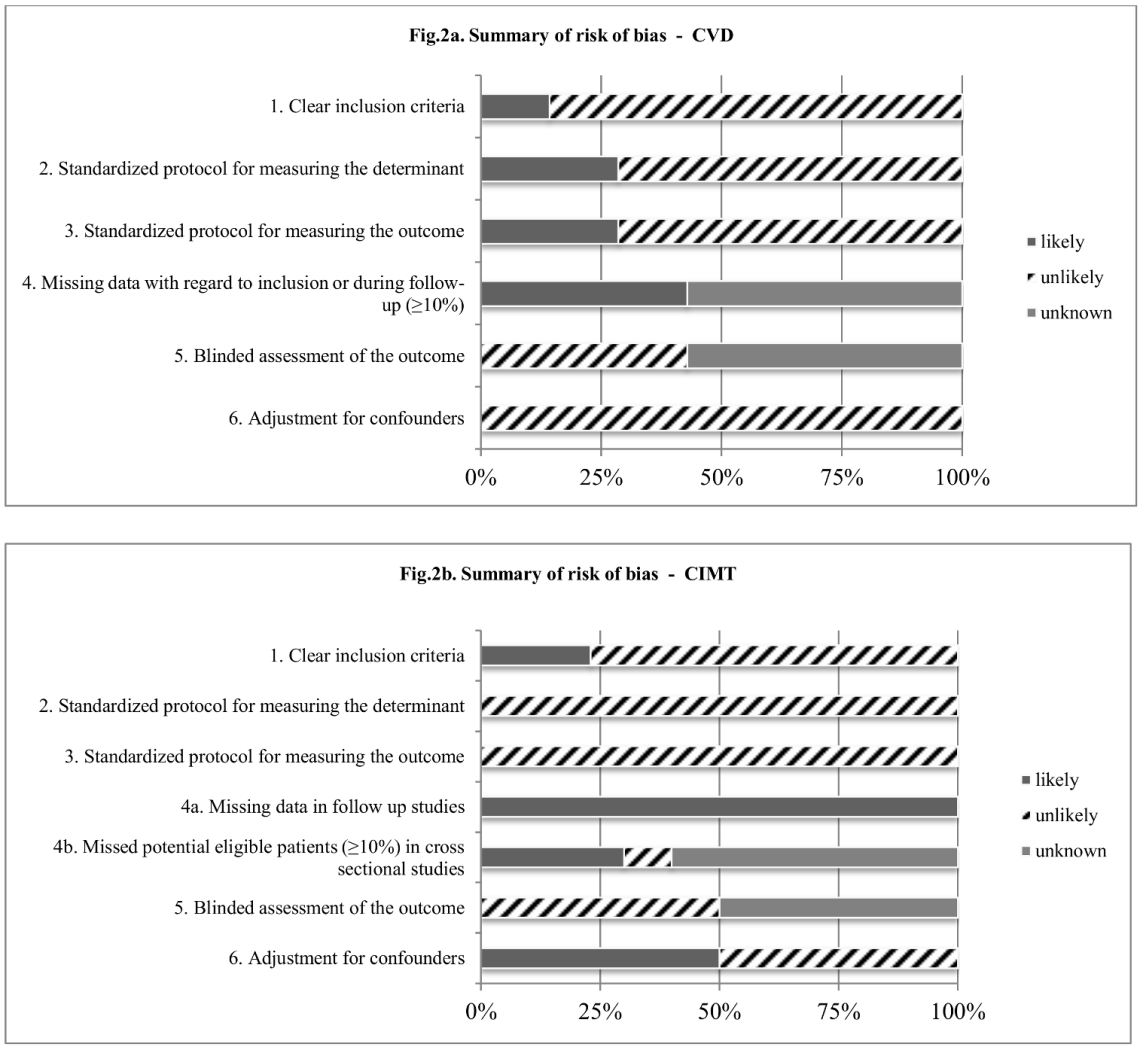
Summary of risk of bias.

All studies were appraised for eight items (Fig 2, S2 Table). The criteria ‘homogeneous moment of inclusion’ and ‘CIMT protocols’ are not incorporated in the figure, since they could not be categorized as ‘yes’ or ‘no’ due to the different aspects that were covered. S2 Table shows marked heterogeneity with regard to the patient populations included for CIMT studies. Although all studies had a standardized procedure for measuring immune markers and outcome, these procedures were different between studies.

### Cardiovascular disease

**Fig 3:**
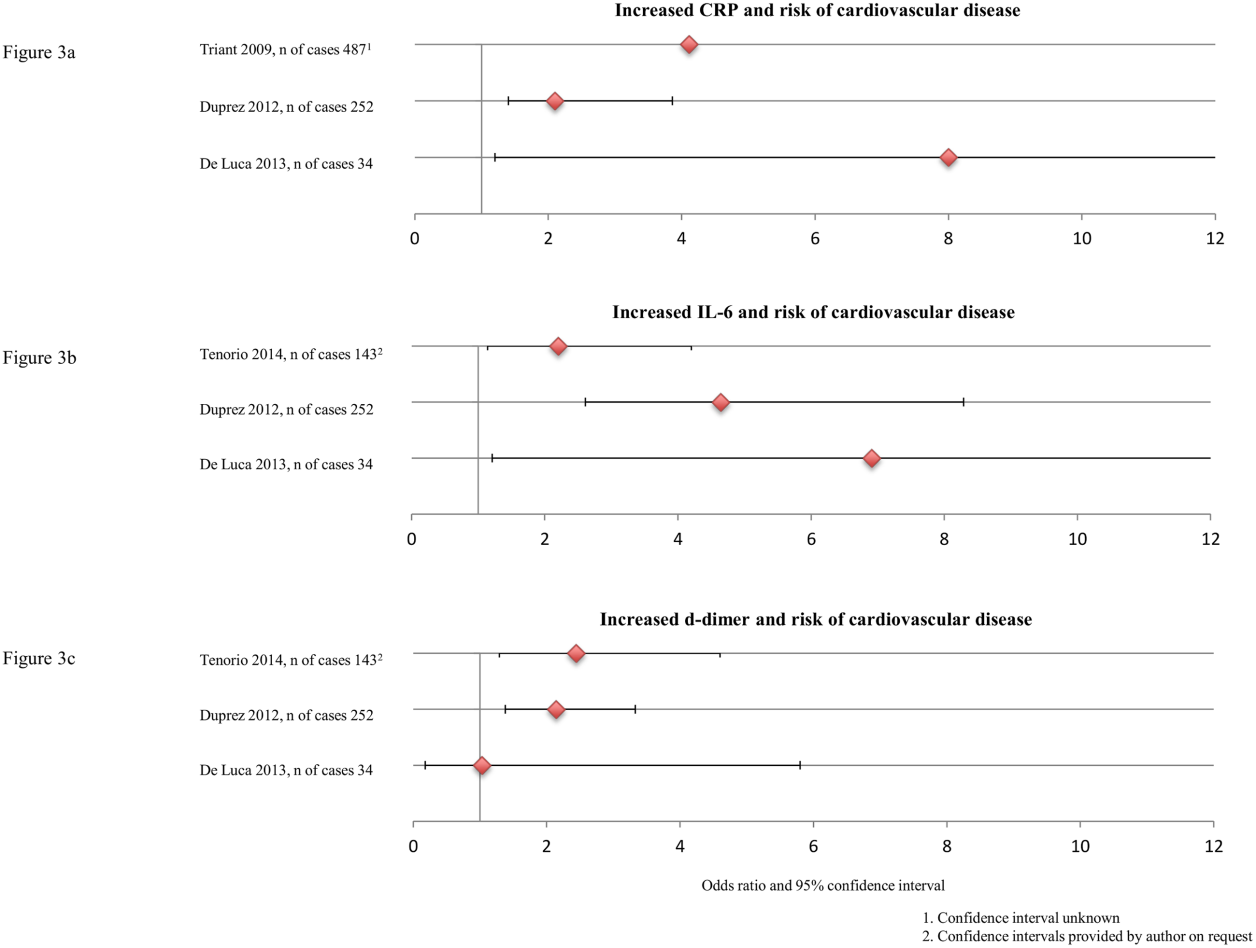
Increased CRP, IL-6 and d-dimer and risk of cardiovascular disease. 1. Confidence interval unknown. 2. Confidence interval provided by author on request.

Most frequently assessed markers across eight studies were CRP (five times), IL-6 (five times), d-dimer (five times) and sCD14 (three times). CRP, IL-6 and d-dimer were assessed four times in relation to the occurrence of CVD [9,51, 52,54,56], and one time in relation to fatal versus non-fatal CVD [13]. These markers were found to be significantly associated with the occurrence of CVD in three out of four studies (Fig 3). [9,51,52,54,56] One article[52] could not be included in the figure since no odds ratios were presented. The authors did not find a relation between CRP, IL-6 and CVD, but they found an association between d-dimer and CVD; it was increased at both 4 months and 2 years prior to events.

Nordell and colleagues[13] used fatal versus non-fatal CVD as outcome. CRP showed no relation, but an increase in IL-6 or d-dimer increased the risk of a fatal CVD, odds ratio and 95% confidence interval for highest versus lowest tertile at baseline were 2.62 (1.26–6.46) and 2.70 (1.27–5.75) respectively. sCD14 was not associated with CVD in any of the three studies.[52,54,58] All other markers (n = 32) were assessed less than three times.

### Carotid intima-media thickness

In studies using CIMT as the endpoint, the most frequently studied inflammation markers were CRP (23 times), interleukin-6 (IL-6) (13 times) and soluble vascular cell adhesion molecule-1 (sVCAM-1) (10 times).

#### C-reactive protein

**Fig 4:**
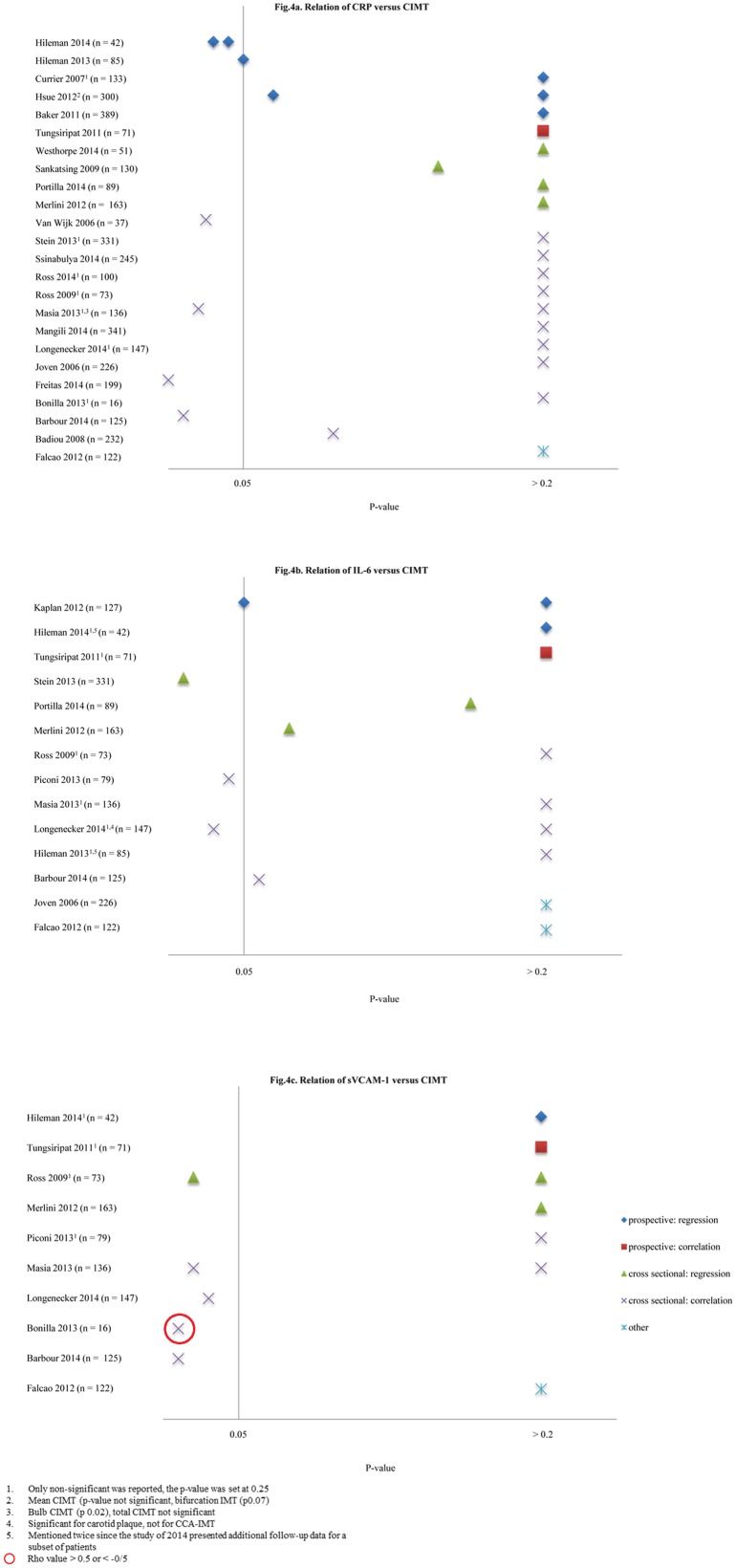
Relation of CRP, IL-6 and sVCAM-1 versus CIMT. 1. Only non-significant was reported, the p-value was set at 0.25, 2. Mean cIMT (p-value not significant, bifurcation IMT p0.07), 3. Bulb CIMT (p0.02), total CIMT not significant, 4. Significant for carotid plaque, not for CCA-IMT, 5. Mentioned twice since the study of 2014 presented additional follow-up data for a subset of patients, Rho value >0.5 or < -0.5.

Fig 4a shows the results of all studies addressing the relation between CRP and CIMT. Four out of seven significant results were calculated using correlation coefficients.[37,39,48,50] The correlations, however, were weak; the highest Rho value was 0.33,[37,48] and were not confirmed in a regression analysis in in two out of four studies.[48,50]

Six studies, describing five patient populations, were prospective with a follow-up duration ranging from 48 to 144 weeks.[19,20,23,30,32,33] The methods that were used to assess the relation between CRP and CIMT differed, varying from a change in CRP versus CIMT progression in a follow-up period of 48 weeks[34], to the association of the baseline level of CRP and CIMT progression in a follow-up period of 48 to 96 weeks[19,20] to the association of the increase of CRP at baseline (in units or doubling of the normal value) versus CIMT increase in millimeters per year[30,59].

The cohort described by Hsue and colleagues[30] showed a significant association between a two-fold increase in CRP at baseline and IMT in univariate analysis, but this association disappeared in multivariable analysis (data not shown).[24,25]

When comparing outcomes from studies including only ART-treated patients (n = 10) [18,33,37,40,43–45,48–50] and studies including only ART-naïve patients (n = 4)[19,20,36,47], no differences were present.

Only one out of eight studies including patients with a suppressed viral load[33,37,40,42–45,49] found a positive correlation[37].

#### Interleukin-6

IL-6 was assessed in 13 studies [10,15,18–20,33,34,39–43,47,50] (Fig 4b), six studies only mentioned that the association was non-significant.[18–20,33,39,43]

Of the prospective studies only Kaplan and colleagues[10] reported a positive association of IL-6 with CIMT in a subset of 81 out of 127 patients. However, the association was very modest (3.1 micrometers CIMT difference per 10% increase in biomarker, 95% CI -0.1–6.3, p 0.05) and only seen following ART initiation.

In a cross-sectional analysis on ART-naïve HIV infected adults, Stein and colleagues[47] found a significant association between IL-6 and carotid lesions (OR 2.1, 95% CI 1.2–3.4), but not for other CIMT segments. Two cross-sectional studies reported a statistically significant but very weak correlation (maximum Rho value 0.22).[18,41]

#### Soluble Vascular Cellular Adhesion Molecule

Ten studies addressed the relation between sVCAM-1 and CIMT (Fig 4c).[18,20,33,34,36,39–41,43,50] In the two prospective studies, no relation was found.[20,33] Although four positive associations were reported in cross-sectional studies [18,36,39,50] only the study of Bonilla and colleagues[36] showed a relevant association for bulb CIMT (Rho-value 0.66). The other correlations were weak, ranging from 0.22 to 0.28 across different CIMT segments.

#### Other markers

Of the remaining markers, twelve were assessed three times or more and 16 markers were only studied once or twice (Table 2). As shown in the table, the majority of these markers did not appear to be significantly associated with CIMT.

**Table 2:** Relation between immune markers and CIMT.

	Positive association	Negative association	No association
**Inflammation**			
TNF- α	2	1	7
sTNFR-1	1	1	5
sTNFR-2	0		6
sCD14	1^1^		8
sCD163	1^2^		3
MCP-1	2		6
MPO	1		3
LPS	1		3
**Endothelial activation**			
sICAM-1	0		7
**Coagulation**			
d-dimer	1		6
fibrinogen	1		7
tPAI-1	0		3
**Other markers assessed less than 3 times**			
CX3CL1	Interleukin-1β	Interleukin-8	Interleukin-10
soluble Interleukin-2 receptor	Mean malonyldialdehyde (MDA)	Matrix metallopeptidase 9 (MMP-9)	Neopterin
Osteoprotegerin (OPG)	Serum amyloid A (SAA)	serum amyloid P component (SAP)	sE-selectin
soluble receptor for advanced glycation end products (sRAGE)	Receptor activator of nuclear factor kappa-B ligand (RANKL)	vascular endothelial growth factor (VEGF)	Von Willebrand Factor (vWF)

^1^. Positive for yearly rate of change in CIMT versus baseline sCD14, cross-sectionally no association,

^2^. Positive correlation for total CIMT, not for bulb CIMT. *CIMT carotid intima media thickness*

## Discussion

We identified forty articles describing 33 original datasets, that addressed the relation between immune markers and CVD or CIMT in HIV-infected individuals. Increased levels of CRP, IL-6 and d-dimer were associated with an increased risk of CVD. Data did not allow calculation of the average effect size or prognostic value for any of the markers. No clear conclusion can currently be drawn for any of the markers assessed in relation to CIMT. This reflects, among other reasons, the heterogeneity in patient populations, cross-sectional nature of most studies and the variability in methods of data analysis.

The finding that levels of CRP, IL-6 and d-dimer are related to CVD is in line with findings in the general population and in populations with other chronic inflammatory conditions like psoriasis and rheumatoid arthritis.[60–66]

Given this evidence, one would expect a positive association between inflammatory markers and CIMT as well. In a recent meta-analysis of individual patient data in the general population [67], a significant relation between CRP and fibrinogen and CIMT at baseline was indeed found. However, none of these markers were longitudinally associated with CIMT or CIMT progression after adjustment for classic cardiovascular risk factors, perhaps reflecting the relative healthy population and a short follow-up (mean of 3.9 years).

Baldassarre and colleagues[68] conducted a systematic review on the relation of immune makers to CIMT in the general population. They reported a significant association between CRP and fibrinogen in relation to CIMT based on a Fisher exact test since it was not possible to perform a formal meta-analysis due to the heterogeneity in ultrasound methodologies and statistical approaches. A Fisher exact test can be used to assess whether or not the number of studies reporting a relation between two determinants is larger than expected under the null hypothesis of no association. When using the Fisher exact test, we similarly found an association between CRP and CIMT (p 0.03), but the use of this test can be questioned. First, as results are simply categorized as ‘positive’ or ‘negative’, depending on the p-value, no between-study differences were taken into account. Second, most positive associations were found by correlation analysis. A positive association, however, does not mean that there is indeed a real association given that a very low correlation coefficient can be statistically significant if numbers are large enough.

CRP is lower in individuals with chronic HCV infection.[69] As chronic HCV infection is common among HIV-infected individuals, this might be a confounding variable, explaining why no relation between CRP and CIMT was observed.

For other, less frequently investigated, markers, conclusions on the association with CIMT are even more difficult. We did show a relation between immune activation and CVD, therefore a similar relation for CIMT was expected. The inconclusive results for CIMT are likely due to the already mentioned between-study heterogeneity and the scarcity of prospective data. Besides, only a few markers are analysed in depth as a result of the enormous variety in marker choice, not allowing for firm statements with regard to the majority of markers. From a pragmatic point of view and with an eye on the costs of marker measurement (approximately £5.50/sample), future research should first explore the value of well-established biomarkers, before embarking on a fishing expedition to find any immune-marker ‘associated’ with CIMT.

### Strengths and limitations

To our knowledge this is the first review that provides a full overview of immune-markers in relation to CVD and CIMT in HIV-infected patients. We used a systematic approach covering all available evidence from 1996 onwards, after the initiation of HAART, to July 2014. Since this review directly focuses on the role of immune-markers, it provided a clear, global overview of the current knowledge.

To appreciate the results some limitations need to be mentioned. First, across studies there was a marked heterogeneity in study population, design and methodology of data analysis, limiting the possibilities for a clear summary of outcome data. Second, the vast majority of studies were cross-sectional rather than prospective. Third, the only common measure of association in studies assessing CIMT was a p-value. For reasons of comparability we decided to present the p-value, although we recognize the dependence on the sample size and the lack of parameter estimates. Forth, co-infection with hepatitis C was not taken into account, which may have led to an underestimation of our results. Finally, we did not take into account the differences in protocols for the assessment of CIMT nor the relation of markers for the diverse CIMT segments (common, bulb, internal). By regarding all segments as being the same we might have overlooked a specific association.

### Recommendations

To obtain reliable information on the prognostic value of inflammatory markers in relation to CVD in HIV-infected-patients research addressing hard CVD outcomes in follow-up studies is needed. Currently some large prospective studies are undertaken, like the REPRIEVE trial[70] and the PURE study [71] that will provide data addressing the relation between inflammation, cardiovascular diseases and HIV infection.

As long as these data are not available CIMT could be used as a surrogate, preferably prospectively and with extended follow-up, and choice of immune-markers should focus on a selective set of markers. Furthermore, studies should be optimized with regard to definition of patient population, data-analysis and reporting. Finally, for reasons of comparability, it would be advisable to standardize the CIMT protocols and the definitions of outcomes.

## Conclusion

This review gives an overview of available evidence regarding the role of inflammation in relation to CVD and CIMT in HIV-infection. Although an association between three immune-markers (CRP, IL-6 and d-dimer) and CVD was observed, no consistent relation with CIMT could be detected for any of the immune-makers. This might reflect the heterogeneity of the CIMT-studies and the lack of adequate prospective data. In view of the costs and interpretability, the search for immune markers ‘associated’ with CIMT in cross-sectional studies should be reconsidered. Future research should aim to be of prospective design, utilizing standardized approaches for the selection of participants, immune markers and assessment of the outcome.

## Supporting Information

S1 File PRISMA checklist. (DOC)

S2 File Prospero upload version. Review protocol. (PDF)

S1 Table Baseline table. (DOCX)

S2 Table Critical appraisal table. (DOCX)
