# Supplementary material for: Pro-Inflammatory Markers in Relation to Cardiovascular Disease in HIV Infection. A Systematic Review
Source: PLoS One. 2016 Jan 25;11(1):e0147484. doi: 10.1371/journal.pone.0147484 (PMC4726827; doi:10.1371/journal.pone.0147484)
Supplement: S2 File — (PDF) [file pone.0147484.s002.pdf]

PROSPERO International prospective register of systematic reviews

Review title and timescale

1 Review title
Give the working title of the review. This must be in English. Ideally it should state succinctly the interventions or exposures being reviewed and the associated health or social problem being addressed in the review.
Pro-inflammatory markers in relation to cardiovascular disease in HIV infection

2 Original language title
For reviews in languages other than English, this field should be used to enter the title in the language of the review. This will be displayed together with the English language title.
not applicable

3 Anticipated or actual start date
Give the date when the systematic review commenced, or is expected to commence.
02/07/2014

4 Anticipated completion date
Give the date by which the review is expected to be completed.
31/10/2014

5 Stage of review at time of this submission
Indicate the stage of progress of the review by ticking the relevant boxes. Reviews that have progressed beyond the point of completing data extraction at the time of initial registration are not eligible for inclusion in PROSPERO. This field should be updated when any amendments are made to a published record.

The review has not yet started ×

Review stage	Started	Completed
Preliminary searches	Yes	No
Piloting of the study selection process	No	No
Formal screening of search results against eligibility criteria	No	No
Data extraction	No	No
Risk of bias (quality) assessment	No	No
Data analysis	No	No

Provide any other relevant information about the stage of the review here.

Review team details

6 Named contact
The named contact acts as the guarantor for the accuracy of the information presented in the register record.
Alinda Vos

7 Named contact email
Enter the electronic mail address of the named contact.
a.g.vos-8@umcutrecht.nl

8 Named contact address
Enter the full postal address for the named contact.
Julius Center UMC Utrecht Heidelberglaan 100 PO Box 85500 3508 GA Utrecht

9 Named contact phone number
Enter the telephone number for the named contact, including international dialing code.
0887568011

10 Organisational affiliation of the review
Full title of the organisational affiliations for this review, and website address if available. This field may be completed as 'None' if the review is not affiliated to any organisation.

Julius center for Health Sciences and Primary Care, University Medical Center Utrecht

Website address:
www.juliuscentrum.nl

11 Review team members and their organisational affiliations

Give the title, first name and last name of all members of the team working directly on the review. Give the organisational affiliations of each member of the review team.

Title	First name	Last name	Affiliation
Miss	A.G.	Vos	Julius Center UMC Utrecht
Miss	N.	Idris	Julius Center UMC Utrecht
Dr	R.E.	Barth	UMC Utrecht, department of infectiology
Dr	K.	Klipstein-Grobusch	Julius Center UMC Utrecht
Professor	D.E.	Grobbee	Julius Center UMC Utrecht

12 Funding sources/sponsors

Give details of the individuals, organizations, groups or other legal entities who take responsibility for initiating, managing, sponsoring and/or financing the review. Any unique identification numbers assigned to the review by the individuals or bodies listed should be included.

No funding

13 Conflicts of interest

List any conditions that could lead to actual or perceived undue influence on judgements concerning the main topic investigated in the review.

Are there any actual or potential conflicts of interest?

None known

14 Collaborators

Give the name, affiliation and role of any individuals or organisations who are working on the review but who are not listed as review team members.

Title	First name	Last name	Organisation details

Review methods

15 Review question(s)

State the question(s) to be addressed / review objectives. Please complete a separate box for each question.

To assess whether and which pro-inflammatory markers are associated with cardiovascular disease in HIV infection This relation will be further specified by looking at subgroups of patients - Patients on antiretroviral therapy (ART) versus patients not on ART - Patients with the metabolic syndrome versus patients without the metabolic syndrome (defined by using the criteria of NCEP ATP3 2005)12 - Patients with an early stage of HIV infection versus an advanced stage of HIV infection (measured by CD4 level or viral load) (stage 1 versus stage 2-4)

16 Searches

Give details of the sources to be searched, and any restrictions (e.g. language or publication period). The full search strategy is not required, but may be supplied as a link or attachment.

MEDLINE Restriction: search in title/abstract. Language other than English and studies before 1996 will be excluded. EMBASE Restriction: search in title/abstract, no MEDLINE. Language other than English and studies before 1996 will be excluded. Cochrane Restriction: search in title/abstract. Language other than English and studies before 1996 will be excluded.

17 URL to search strategy

If you have one, give the link to your search strategy here. Alternatively you can e-mail this to PROSPERO and we will store and link to it.

http://www.crd.york.ac.uk/PROSPEROFILES/10516_STRATEGY_20140603.pdf

I give permission for this file to be made publicly available

No

18 Condition or domain being studied

Give a short description of the disease, condition or healthcare domain being studied. This could include health and wellbeing outcomes.

HIV infected patients

19 Participants/population

Give summary criteria for the participants or populations being studied by the review. The preferred format includes details of both inclusion and exclusion criteria.

Inclusion: HIV infected human beings, aged 18 years or older

20 Intervention(s), exposure(s)

Give full and clear descriptions of the nature of the interventions or the exposures to be reviewed

Determinant: pro-inflammatory markers. Markers of inflammation are defined as markers that could be measured in plasma or serum. Exclusion: cellular blood components (i.e. lymphocyte subsets) and genetic markers

21 Comparator(s)/control

Where relevant, give details of the alternatives against which the main subject/topic of the review will be compared (e.g. another intervention or a non-exposed control group).

n.a.

22 Types of study to be included initially

Give details of the study designs to be included in the review. If there are no restrictions on the types of study design eligible for inclusion, this should be stated.

Inclusion: Only original research: Observational cohort studies Cross-sectional studies Case-control studies Intervention studies including the domain: human beings aged 18 years or older including one ot the outcomes: cardiovascular disease or a surrogate marker of cardiovascular disease. Exclusion: animal studies Case series under 10 cases Studies published before 1996

23 Context

Give summary details of the setting and other relevant characteristics which help define the inclusion or exclusion criteria.

n.a.

24 Primary outcome(s)

Give the most important outcomes.

cardiovascular disease: myocardial infarction, cardiac death, stroke

Give information on timing and effect measures, as appropriate.

25 Secondary outcomes

List any additional outcomes that will be addressed. If there are no secondary outcomes enter None.

surrogate markers of cardiovascular disease 1) intima media thickness (carotid or femoral) 2) pulse wave velocity 3) ankel brachial index 4) coronary calcium score 5) flow mediated dilation

Give information on timing and effect measures, as appropriate.

26 Data extraction, (selection and coding)

Give the procedure for selecting studies for the review and extracting data, including the number of researchers involved and how discrepancies will be resolved. List the data to be extracted.

Selection of studies will be done by a three-step wise model. Firstly, all identified records will be screened on title and abstract using above mentioned inclusion criteria. Secondly, of the remaining abstracts full text reports will be read to determine eligibility. This will be done by 2 authors independently (AV and NI) using the following inclusion criteria: 1. assessment of at least one pro-inflammatory marker 2. relation between inflammatory marker and CVD, expressed in a numeric value (e.g. risk, probability data, mean, difference) In case only an abstract is available or outcome data are insufficient or unclear authors will be contacted for additional information. Thirdly, references and citations of the selected articles will be checked for additional eligible articles. Differences in inclusion will be discussed in a consensus meeting. In case no consensus can be reached, a third reviewer (RB) will be consulted. In case of persistent differences a consensus meeting with al authors will be organized where a final decision will be taken. Data extraction will be done independently by 2 authors (AV, NI) using a predefined data extractionform, including the

following items: Year of publication, study design, duration of follow up, number of patients, country and setting will be recorded. For the different subgroups the following data will be extracted: age, sex, HIV status, ART use, duration of HIV infection, duration of ART use, cardiovascular risk factors, inflammatory parameters measured, outcome and outcome measures.

27 Risk of bias (quality) assessment

State whether and how risk of bias will be assessed, how the quality of individual studies will be assessed, and whether and how this will influence the planned synthesis.

Selected studies will be critically appraised to assess the risk of bias. The Cochrane Collaboration's tool for assessing risk of bias will be used, adjusted for descriptive research by using the STROME-ID guideline. The main focus will be on the risk of selection bias, detection bias and attrition bias. A predefined critical appraisal form will be used. Critical appraisal will be done by 2 authors independently (AV, NI).

28 Strategy for data synthesis

Give the planned general approach to be used, for example whether the data to be used will be aggregate or at the level of individual participants, and whether a quantitative or narrative (descriptive) synthesis is planned. Where appropriate a brief outline of analytic approach should be given.

Study designs and pro-inflammatory parameters included will be very heterogeneous. If data allow a meta-analysis will be performed and results will be presented in a forest plot. If this is not the case a quantitative synthesis will be performed. Results will be grouped by outcome and type of inflammatory marker. Besides this, there will be stratification by study design: cross-sectional versus longitudinal.

29 Analysis of subgroups or subsets

Give any planned exploration of subgroups or subsets within the review. 'None planned' is a valid response if no subgroup analyses are planned.

In case of sufficient data, outcomes will be reported per pre-defined subgroup of patients (antiretroviral therapy yes or no, metabolic syndrome yes or no, early stage or advanced stage of HIV)

Review general information

30 Type of review

Select the type of review from the drop down list.

Prognostic

31 Language

Select the language(s) in which the review is being written and will be made available, from the drop down list. Use the control key to select more than one language.

English

Will a summary/abstract be made available in English?

Yes

32 Country

Select the country in which the review is being carried out from the drop down list. For multi-national collaborations select all the countries involved. Use the control key to select more than one country.

Netherlands

33 Other registration details

List places where the systematic review title or protocol is registered (such as with he Campbell Collaboration, or The Joanna Briggs Institute). The name of the organisation and any unique identification number assigned to the review by that organization should be included.

none

34 Reference and/or URL for published protocol

Give the citation for the published protocol, if there is one.

n.a.

Give the link to the published protocol, if there is one. This may be to an external site or to a protocol deposited with CRD in pdf format.

I give permission for this file to be made publicly available
Yes

35 Dissemination plans
Give brief details of plans for communicating essential messages from the review to the appropriate audiences.
The results will be presented in a paper, with the intent to publish the review in an international journal.

Do you intend to publish the review on completion?
Yes

36 Keywords
Give words or phrases that best describe the review. (One word per box, create a new box for each term)
HIV

inflammation

markers of inflammation

cardiovascular disease

surrogate markers of cardiovascular disease

37 Details of any existing review of the same topic by the same authors
Give details of earlier versions of the systematic review if an update of an existing review is being registered, including full bibliographic reference if possible.
n.a.

38 Current review status
Review status should be updated when the review is completed and when it is published.
Ongoing

39 Any additional information
Provide any further information the review team consider relevant to the registration of the review.

40 Details of final report/publication(s)
This field should be left empty until details of the completed review are available.
Give the full citation for the final report or publication of the systematic review.
Give the URL where available.
