# Supplementary material for: Pro-Inflammatory Markers in Relation to Cardiovascular Disease in HIV Infection. A Systematic Review
Source: PLoS One. 2016 Jan 25;11(1):e0147484. doi: 10.1371/journal.pone.0147484 (PMC4726827; doi:10.1371/journal.pone.0147484)
Supplement: S1 Table — (DOCX) [file pone.0147484.s003.docx]

| **Author** | **(Design &) enrollment period** | **Duration of follow up** | **Number**  **of HIV-positive patients** | **Country** | **Ethnicity** | **%** ♂ | **Age** | **Years since HIV diagnosis** | **CD4 count** | **Nadir CD4 count** | **VL** | **BMI (kg/m2)** | **Current smokers** | **ART + (%)** | **Tx regimen** | **Duration of ART**  **(months)** | **Markers assessed** | **Stored sample used** | **Time of outcome with regard to biomarker** | **Methods** |
| --- | --- | --- | --- | --- | --- | --- | --- | --- | --- | --- | --- | --- | --- | --- | --- | --- | --- | --- | --- | --- |
| **Cardiovascular events** | | | | | | | | | | | | | | | | | | | | |
| De Luca 2013 | Nested case-control study. Enrollment  since 1997 | Cases nd Controls ≥5yrs. | 35 cases (with CVD event), 74 controls (no CVD event) | Italy | nd | 97 / 89 | 47/45 | nd | 550/525 | nd | 2.1/1.7 log_10_ cp/ml | 23.7/23.1 | Distribution of matching variable smokers/diabetics (3;4) | 100 | nd | NRTI 51/121  NNRTI 3/30  PI 26/52 | hscRP, d-dimer, p-selectin, IL-6, t-PA, PAI-1 | Yes | Most recent sample:3 months/8 months  Older sample: nd | Nephelometry (hsCRP)  Immunoturbidimetric assay (d-dimer)  ELISA (other)  plasma |
| Ford 2010  Appendix 1. Baseline table | Nested case control within NIAID  1995-2009 | 8.2 / 8.6 years | 52 cases  104 controls | USA | African-American 19.2% / 14.4% | 98 / 98 | 50.8 / 50.8 | 13.4/ 14.0 | nd | 209 / 229 | Peak 4.3/5.4 cp/mL | 25.6/ 25.6 | 49.0 / 25.0 | 100 | nd | 107/101  PI exposure  44/48 | CRP, IL-6, d-dimer.  sCD14, sFT,  GM-CSF, INF-ϒ, IL-1β, IL-10, IL12p70, IL-2, Il-8, TNF-α, eotaxin, eotaxin-3, IP-10, MCP-1, MCP-4, MDC, MIP-1β, TARC, SAA, VCAM-1, ICAM-1, CRP, MPO, TIMP-1, TNRF-II | Yes | 4.5 months and 21.6 months prior to event | d-dimer ELISA (Vidas)  sCD14, sFT ELISA (R&D)  Other ELISA multiplex kits  plasma |
| Knudsen 2013 | Nested case control study within Danish HIV Cohort Study  1998-2008 | 1.3 yr (medium, between first plasma sampel and event) | 55 cases  182 controls | Denmark | White 93 / 94% | 91 / 92% | 49/50 | 10 /10 | 496 / 547 | nd | <400cp/mL  80/89% | nd | 96/97 | 100 | NRTI 100%  Abacavir 56/39%  NNRTI 73/58%  PI 87/84% | 72/72 | sCD163 | Yes | 4 samples:  1: before start of ART  2: 3 months after start of ART  3: one year before event  4: sample before event  52 days to event | ELISA  plasma |
| Nordell 2014 | Retrospective cohort study of SMART, ESPRIT, SILCAAT | 5 yrs (median) | 288 cases: 74 fatel, 214 non-fatal  8766 non cases | Resp 33, 25 and 11 countries | Black resp 23/16/19% | 86 / 92 / 78 | 48 / 49/ 42 | nd | 409/469/487 | nd | <500cp/mL 74/77/77 | 24.0/ 24.3/ 24.3 | 53/52/41%^3^ | 100 | 81/87/84 in SMART, remaining 100% | nd | hsCRP, IL-6, d-dimer | Yes | Baseline samples | hsCRP ELISA (nephelometer, R&D)  IL-6 ELISA  d-dimer ELISA (Sta-R and VIDAS)  plasma |
| Sandler 2010 | Nested case control within SMART | Median of 16 months | 120 cases, 238 controls | Enrollment in 33 countries | White 58.3/61.8  Black 42 / 38% | 81 / 81 | 49 / 48 | nd | 607/638 | 209/241 | ≤400 cp/mL (%) 66/59 | 25.2/25.6 | 55.0 / 37.4 | 100 | nd | 48 months (median among all subjects) | I-FABP, LPS, sCD14, EndoCAb | Yes | Plasma samples taken at baseline | Elisa  sCD14 (R&S systems)  EndoCAb, I-FABP (Cell sciences)  LPS (Limulus assay)  Plasma |
| Tenorio 2014 | Case control study ACTG ALLRT participants | 2.9 yrs (median, after ART initiation) | 143 cases (all causes), 315 controls | USA | White 52/48%W  Black 36/28%  Hispanic 11/20% | 85 / 85 | 46/44 | nd | 208/221 | nd | 4.8 / 4.8  Log_10_ cp/mL | 0.92 / 0.92 *(waist to hip ratio, median)* | 75 / 55* | 100 /100 | nd | 2.9 yrs *(median, same as follow up duration)* | IL-6, sCD14, IFN-y, IP-10, sTNFR-1 and II, d-dimer | yes | Samples at baseline (before start of ART), 1 year after start of ART, and pre-event | R&D systems: all, except d-dimer.  D-dimer: Diagnostica Stago  Plasma |
| Triant 2009 | Retrospective cohort study within Partners HealthCare System 1997-2006 | 6.0 yrs (mean) | 487 HIV+ cases  69870 HIV- cases | USA | White 58%  Nonwhite 38% | 62.8% | ≥55 yrs 24.7% | nd | > CRP: 43% <200  CRP =  28% <200 | nd | > CRP: undetectable in 34%  CRP =: undectable in 38% | nd | 57%* | nd | NNRTI 43%  PI: > CRP 67%  CRP= 39% | nd | (hs)CRP | no | 199 days (median) | Both standard and high sensitivity assays  Plasma/serum: nd |
| **Studie describing the same databases as one of the above mentioned studies, but with additional information** | | | | | | | | | | | | | | | | | | | | |
| Duprez 2012 (partly double with Nordell 2014) | Retrospective cohort study of SMART database. Inclusion between 2002-2006 | Min. 18 median 29 months | 252 cases  4846 non-cases | Enrollment in 33 countries | Black 40 / 29% | 81 / 74% | 49/43 | nd | 579/600 | 236/252 | ≤400cp/mL 68 / 71% | 25.7/25.0 | 52/41% | 100 | nd | nd | hsCRP, IL-6, d-dimer | Yes | Median 29 months (same as follow up time) | hsCRP nephelometer  IL-6 ELISA (R&D)  d-dimer immunoturbidometer  plasma |
| **Carotid intima-media thickness; longitudinal** | | | | | | | | | | | | | | | | | | | | |
| Baker  2011 | Prospective cohort study March 2004 – June 2006  Measurement at T0 and T1 | 2 yrs | 389 | USA | White, non- Hispanic 26%  Black, non hispanic 12% | 77 | 42 | 4.7 | 485 | 215 | VL <400cp/ml 72% | 26 | 38% | 78 | NNRTI 39%  PI 35%  Abacavir 23% | 32 | hsCRP | yes | = | Immunoturbidimetry  Plasma |
| Currier 2007 | Prospective matched cohort  Triads of 1)HIV and PI>2yrs  2) HIV without PI use  3) HIV uninfected February 2001 – May 2002 | 144 weeks | 133 | USA | White 76%  Black 4%  Hispanic 16% | 90 | 42 | nd | 530/481  (PI vs non PI group) | nd | <400cp/ml  75/69%  (PI vs non PI group) | nd | 45%^2^ | 97% | PI ±33% | nd | hsCRP | nd | = | nd |
| Hileman 2013 | Matched prospective cohort study July 2008-April 2010 | 48 wks | 85 HIV+  (45 HIV-) | USA | Caucasian 39%  African American 58%  Latino 2% | 78 | 40  (32-47) | 3.3 | 535 | nd | 6916 cp/ml  *RNA* | 27 | 52% | 0 | na | na | hsCRP, IL-6, TNFR-1,2, sVCAM-1, sICAM-1, d-dimer, fibrinogen | yes | = | Nephelometry (hsCRP, fibrinogen)  Turbidometry  (d-dimer); other markers with ELISA  Plasma |
| Hsue 2012 | Prospective cohort study (within the SCOPE cohort)nd | 2.4 yrs | 300 HIV +  (47 HIV- controls) | USA | Caucasian 60%, African American 25%  Latino 10% | 89 | 47  (41-53) | 13 | 434 | 172 | <75 cp/ml 53% | 25 | 69%^2^ | 76 (ever) | NRTI use (ever) 76%  NNRTI use (ever) 48%  PI use (ever) 65% | 60 | hsCRP | nd |  | Dade Behring  Plasma/serum: nd |
| Kaplan  2012 | Matched prospective cohort  (within the WIHS cohort)  1994-1995  (pre-HAART)  2001-2002 | 2,5yrs | 127 HIV+  (127 HIV- controls) | USA | African-American 59%  Latina 24%  White/  Caucasian 17% | 0 | 37  (33-42) | nd | 332 (before start of ART) | nd | 4.4 (log VL)  (before start of ART) | >25  61% | 53% | 100 | PI 53%  NNRTI 35%  NRTI 93% | Started during the study period | sCD14, TNF-a, sIL-2rec, IL-6, IL-10, MCP-1, d-dimer, fibrinogen | yes | 2004-2005  (≠ timing measurement biomarker) | ELISA (sIL-2R, IL-6, sCD14)  Clot-based assay (fibrinogen)  Immunoturbidemetric (d-dimer)  Bead-based immunoassay (MCP-1, TNF-a, IL-10) |
| Tungsiripat  2011 | RCT  (rosiglitazone or placebo)  July 2006- December 2007 | 48 wks | 71  *with lipoatrophy* | USA | White 51% | 83 | 47/52 | 12.2/14.2 | 595/690 | 205/123 | <50cp/ml 80% | 25.3/25.8 | nd | 100 | NNRTI 41%  PI 59% | 114/105 | hsCRP, IL-6, TNF-a, sTNFRI,II, vWF, sVCAM-1, sICAM-1, MPO | no | = | ELISA  Plasma/serum: nd |
| **Carotid intima-media thickness; cross-sectional** | | | | | | | | | | | | | | | | | | | | |
| Badiou  2008 | Sept-dec 2009  c.s. | na | 232 | France | nd | 75 | 41 ±9 | nd | 465 | nd | 2.9±1.1  (log) | 22 | 70% | 80 | 46% PI containing  31%NNRTI | 37±26 | hsCRP | yes | = | Immunoturbidimetric  Plasma/serum: nd |
| Barbour  2014 | Baseline analysis of a cohort study with a follow up of 5 years. Enrollment period: nd | na | 125 | Hawaii | White 55% | 87 | 49.5 | nd | 479 | nd | 69% undetectable | nd | 22% | 100 | nd | nd | CRP, IL-6, IL-8, IL-10, IL-1B, TNF, MPO, MMP-9, tPAI-1, sVCAM-1, sICAM-1, sE-selectin, MCP-1,VEGF, sCD14, SAA, SAP | nd | nd | High sensitivity Milliplex assay – Luminex  Plasma |
| Bonilla^1^  2013 | nd  c.s. | na | 16 LTNP (16 HIV- controls) | USA | White 62.5% | 75 | 42.8 | 13.9 | 586 | 512 | nd | 26.4 | nd | 0 | Na | Na | hsCRP, sVCAM-1, sTNFR-II | yes | nd | ELISA  Plasma/serum: nd |
| Falcão  2012 | 2008-2010  c.s. | na | 122 | Brazil | nd | 61 | 57.4% >40yrs | nd | ≥200 86% | <200 63% | undetectable 41% | Overweight/obesity 40% | 28% | 81 | PI 35% | <5 yrs 57%  >5 yrs 25% | hsCRP, IL-6, TNF-a, IL1B, sVCAM-1, sICAM-1 | yes | nd | nephelometry  Multiplex bead-array assay  Plasma |
| Freitas  2014 | nd  c.s. | na | 199  117 without -, 82 with LD | Portugal | Caucasian | 61/74 | 45/49 | 7.2/8.5 | 503/632 | nd | <50 87/90% | 25/25 | 47/33% | 100 | PI 51/59  NNRTI 50/46  NRTI 97/96 | 68/95 | hsCRP | no | = | ‘commercial kit’,  not further specified  ‘venous blood’ |
| Jeong  2011 | nd  c.s. | na | 76 | Korea | ‘Korean patients’ | 100 | 40.5 | 3.9 | 324 | nd | 1,7 (log VL) | 23.4 | nd | 100 | On PI 57%  On NNRTI 43% | 32 | sRAGE | yes | = | ELISA  Plasma/serum nd |
| Joven  2006(data per quartile MCP-1) | nd  c.s. | na | 226  (384 HIV- individuals) | Spain | Caucasian 100% | 70 | 40 | nd | 369-533 | nd | 2.5-3.1 (log VL) | 18.9-19.7 | 81-88% | 57-75% | PI 27-47%  NNRTI 25-48% | nd | hsCRP, MCP-1 | no | = | hsCRP ‘highly sensity method’  ELISA  Plasma/serum: nd |
| Longenecker  2014 | From march 2011  c.s. | na | 147  HIV RNA <1000 copies/ml | USA | African-American 69% | 78 | 46 | 12 | 613 | 179 | <48 cp/ml 70% | 27 | 63% | 100 | PI use 49% | 64 | hsCRP, IL-6, sTNFR-1, sCD14, sCD163, sVCAM-1, fibrinogen, d-dimer, OPG, RANKL | no | = | Immunonephelometry (CRP, fibrinogen)  Immunoturbidometry (d-dimer)  ELISA (other)  Plasma/serum: nd |
| Mangili 2014  (results stratified by LpPLA2 mass) | January 2002- March 2004  c.s. | na | 341 | USA | White 52%  Black 34% | 75 | 44 | 9.6-10.2 | 385-470 | nd | 2.9-3.6 (log VL) | 26-28 | 49% | 74 (on HAART) | On PI 44% | 25-33 | CRP | yes | Within a 3 month time period | Immunoturbidimetry  Plasma |
| Masia  2013 | nd  c.s. | na | 136 | Spain | Caucasian 97.1% | 99 | 49 | nd | 650 | nd | All: <200cp/ml | nd | 49% | 100 | PI use 43%  NNRTI use 39% | nd | hsCRP, IL-6, TNF-a, sVCAM, sICAM, MCP-1, PAI-1, sCD163, sCD14,  d-dimer,MDA | yes | = | Immunometry (CRP)  HPLC analysis (MDA)  ELISA (other)  Plasma |
| Merlini  2012 | nd  c.s. | na | 163 | Italy | Caucasian 94% | 82 | 48 | 12 | 496 | 210 | Undetectable 100% | 25 | 48% | 100 | PI 56%  NNRTI 34% | 60 | hsCRP, IL-6, TNF-a, sCD14, sVCAM-1,LPS | nd | Not clearly defined | ELISA  LAL kit (LPS)  Plasma |
| Piconi  2013 | nd  c.s. | na | 76  55 ART+  21 ART- | Italy | nd | 100 | 46/43 | 10.3/7.8 | 546/500 | 374/169- | <37cp/ml: all ART treated patients | 24/20 | 64%  (>10 sig/day) | 57 | PI use (months) 50  NNRTI use (months) 33  %: nd | 98 | TNF-a, IL-6, MCP-1, sVCAM-1, sICAM-1, fibrinogen, d-dimer | yes | Not clearly defined | ELISA  Plasma |
| Portilla  2014 | March 2009-Oct 2010  c.s. | na | 89 | Spain | Caucasian 100% | 100 | 42 | 7.8 | 467 | 204 | Undetectable in all ART treated patients | 24.8 | 61% | 84 | PI 44%  NNRTI 40% | 67 | hsCRP, IL-6, TNF-a, sTNFR-I,II, PAI-1 | yes | = | Turbidimetry (hsCRP)  ELISA  Plasma/serum: nd |
| Ross  2009 | nd  c.s. | na | 73, on ART ≥24 weeks  (21 HIV-) | USA | African-American 38%  White 45%  Hispanic 10% | 81 | 48 | 13.5 | 624 | 162 | <50 cp/ml 81% | 26 | 38% | 100 | nd | Cumulative duration of (months):  PI 53  NRTI 96  NNRTI 17 | TNF-a, STNFR-I, II, IL-6, hsCRP, MPO,vWF, sICAM-1, sVCAM-1 | no | = | ELISA  Plasma |
| Ross  2014 | March 2011-August 2012 c.s. | na | 100 | USA | Black 70%  Caucasians 29% | 77 | 47 | 13 | 633 | 199 | Undetectable in 80% | 27 | 62% | 100 | PI 74%  NNRTI 52% | 75 | hsCRP | yes | = | Nephelometry  Plasma/serum: nd |
| Sankatsing  2009 | June 2003 – February 2006  c.s. | na | 130 (same ART regimen for ≥ 2 yr) | The Netherlands | nd | 90 | 46 | nd | nd | nd | Undetectable  100% | 23-24  (PI vs NNRTI use) | 44% | 100 | PI based 48%  NNRTI based 52% | 61/60  (PI vs NNRTI use) | hsCRP | no | Not clearly defined | Immunoturbidimetry  Plasma/serum: nd |
| Ssinabulya  2014 | Febr-Oct 2012  c.s. | na | 245 (ART naïve or treated) | Uganda | nd | 31 | 37 | nd | nd | 124 | nd | 21.5 | 5% | 41 | NNRTI 86%  2nd line ART (PI) 14% | ART al least 7 yrs: 41% (median) | hsCRP | yes | Not clearly defined | ELISA  Plasma (EDTA) |
| Stein  2013 | nd  c.s. | na | 331 | USA | White 44%  Black 32%  Hispanic 20% | 89 | 36 | 0.5 | 349 | nd | 4.5 (log VL) | 25 | 38% | 0 | na | na | hsCRP, IL-6 | yes | = | Nephelometry (hsCRP)  ELISA  Plasma |
| Van Wijk  2006 | nd  c.s. | na | 37  (15 MS+, 22 MS-) | The Nether lands | nd | 100 | 50/47 | 8.5/7.5 | 604/719 | nd | undetectable 80/77% | 24.4/23.6 | 13/14% | 100 | PI 67/68%  NNRTI 33/32%  NRTI 100/100% | 58/50 | hsCRP | no | Not clearly defined | Quantes hs-CRP kit  Plasma |
| Westhorpe  2014 | nd  c.s. | na | 51  (+49 HIV- individuals) | Australia | nd | 98 | 49 | 9.3 | 705 | 232 | <50cp/ml 100% | nd | 39% | 100 | NNRTI 85%  NRTI 100% | nd | hs-CRP, sCD163, sCD14, CX3CL1, MCP-1/CCL2, LPS, neopterin, fibrinogen, d-dimer | yes | Not clearly defined | ELISA (sCD163, neopterin, sCD14, SC3CL1, CCL2)  LAL kit (LPS)  Plasma |
| **Studies describing the same databases as one of the above mentioned studies, but with additional information** | | | | | | | | | | | | | | | | | | | | |
| Hileman 2014  (additional to Hileman 2013) | July 2008-April 2010 Matched prospective cohort study | 96 wks | 42 HIV+  (41 HIV- -) | USA | African American 69% | 69 | 40 | 4.8 | 630 | nd | 4900 cp/ml | 27.3 | 67% | 0 | na | na | hsCRP, IL-6, TNFR-1,2, sVCAM-1, sICAM-1, d-dimer, fibrinogen | yes | = | Nephelometry (hsCRP, fibrinogen)  Turbidometry  (d-dimer)  ELISA  Plasma |
| Hsue  2006  (additional to Hsue 2012) | Cross-sectional (within the SCOPE cohort)  ≥1 year off ART or on stable ART | na | 93  (37 HIV-) | USA | Caucasian 62%, African American 25%  Hispanic 8% | 91 | 48 | 13 | 354 | nd | Undetectable 57% | 25 | 42% | 93.5 | PI 88% | For PI users 48 | hsCRP | no | CIMT within 4 months after immune marker analysis | CardioPhase assay  Plasma/serum: nd |
| Hsue 2009  (additional to Hsue 2012) | Cross-sectional (within the SCOPE cohort) | na | 401  (93 HIV-) | USA | Caucasian ca. 50% | 87 | 48 | 11-15 | 452 | 70-491 | <75 cp/ml in 53.1% | nd | 66%^2^ | 92 | nd | 70 (ART responders) vs 59 (non-responders) | hsCRP | no | nd | Dade Behring  Plasma/serum: nd |
| Kelesidis  2012  2013  (additional to Currier 2007) | nd  Historical analysis of a prospective matched cohort study | 3 yrs | 55  (36 HIV negative -individuals) | USA | White non Hispanic 76%  Hispanic 19% | 92 | 41 | nd | 488 | 20 | <50cp/ml 84% | 24.7 | 25.5/24.2 (HIV/PI vs HIV/non PI) | ≥94.5 | Ritonavir use (any) 16%  NRTI use (any) 57%  NNRTI use (any) 31% | 39 | RANKL, OPG, RANKL/OPG axis  sCD14, LPS | yes | = | ELISA  Pyro Gene rFactor C assay (LPS)  Plasma/serum: nd |
| Parra  2010 (additional to Joven) | Nd  c.s. | na | 155  (results grouped on base of atherosclerosis and FRS) | Spain | nd | 57 | 36-47 | 6.8-7.9 | 286-364 | nd | <40cp/ml 68% | 18.8-19.8 | 78% | nd | nd | On NRTI’s 58-65  On PIs 27-30  On NNRTIs 9-11 | CRP, IL-6, MCP-1 | no | Not clearly defined | High sensitivity method (CRP)  Immunoturbidimetry (IL-6)  ELISA |
| 1. Case control, but regarded as c.s. since alone the HIV+ group was used  2. Ever smokers  3. Data only from SMART database  * Current or past / ever  ACTG: AIDS clinical Trails Group, ART: antiviral therapy, cART: combination ART, c.s.: cross-sectional, EndoCAb: endotoxin core igM antibody, FRS: Framinham Risk Score, I-FABP: Intestinal fatty acid binding protein  LD: lipodystrophy, LTNP: long term non-progressors, na: not applicable, nd: no data, MS: metabolic syndrome, NNRTI: non-nucleoside reverse transcriptase inhibitor, NRTI: nucleoside reverse transcriptase inhibitor,  PI: protease inhibitor | | | | | | | | | | | | | | | | | | | | |
