# Supplementary material for: Pro-Inflammatory Markers in Relation to Cardiovascular Disease in HIV Infection. A Systematic Review
Source: PLoS One. 2016 Jan 25;11(1):e0147484. doi: 10.1371/journal.pone.0147484 (PMC4726827; doi:10.1371/journal.pone.0147484)
Supplement: S2 Table — (DOCX) [file pone.0147484.s004.docx]

| **Study** | **Clear inclusion criteria** | **Homogenous moment of inclusion/how were patients included** | **Standardized protocol for measuring the determinant** | **Standardized protocol for measuring the outcome** | **Missing data with regard to inclusion or follow-up** | **Blinded measurement of determinant / outcome** | **Outcome** | **Adjustment for confounders** |
| --- | --- | --- | --- | --- | --- | --- | --- | --- |
| **Cardiovascular events** | | | | | | | | |
| De Luca 2013 | Yes | Patients of I.Co.N.A and CUSH cohort. Incl: age 35-69yrs, no hx of CVD, no inflammarory disorder. Cases: CVD event, on ART, ≥1sample available before CVD event. Matched based on history of diabetes and smoking status. | No (some samples were initially frozen by -20 degrees, and later on -80 degrees) | No. Definition not clearly specified | Number of potential eligible cases not addressed | Yes | major CVD (acute myocardial infarction; stable or unstable angina or were undergoing myocardial revascularization procedures) | Adjusted for sampling time and –year, HIV related factors, type of ARVs, cholesterol, hepatitis B or C |
| Ford 2010 | No | Participants from the National Institute of Allergy and Infectious Diseases (NIAID). HIV+ matched 2:1 based on HIV+, gender, age, enrollment date | Yes | Yes | Number of potentially eligible patients not addressed | nd | CVD: acute MI, silent MI, coronary revascularization, ACS, CVA, PAD, CV death | Multiple analysis including all significant variables in univariate analysis (p <0.05) |
| Knudsen 2013 | Yes | Patients from the Danish HIV Cohort Study with an ICD-10 diagnosis of IHD. Matched 1:4 to controls without CVD, based on age, duration of ART, gender, smoking | Yes | Yes | Number of potentially eligible patients not addressed | nd | Ischemic Heart disease, based on ICD-10 codes | Adjusted for HIV RNA, abacavir, NNRTIs, PIs. |
| Nordell 2014 | Yes | Patients from the SMART trial, ESPRIT, SILCAAT trial.  HIV+, CD4 resp ≥350cells/mm^3^, >300cells/mm3, 50-299cells/mm3 | Yes | Yes | 7.3% loss to FU  Potential eligible pts:  13.4% not included; no blood available (11%), history of CVD at entry (2.4%) | Yes | CVD events: deaths attributed to CVD, unwitnessed deaths not otherwise explained, nonfatal MI or stroke, CAD requiring surgery | Adjusted for age, gender, study, time between biomarker and event, race, BMI, HIV RNA, CD4, earlier AIDS event |
| Sandler 2010 | Yes | Patients with a CVD event from the SMART cohort. Age ≥13 yrs, CD4 count >350cells/mm^3^. Matched 1:2 to controls based on age, sex, country, date of enrollment (≤3 months) | Yes | No (Not clearly stated. Definition of diseases not given) | potential eligible participants: 120/142 cases analyzed, based on available blood samples | Yes | CVD event: myocardial infarction, stroke, coronary artery disease, congestive heart failure, peripheral vascular disease, deaths attributed to CVD, unwithnessed deaths | Adjusted for matching factors, HIV-related factors, CV risk factors, Hep B and C, Tx arm. 2^nd^ model: corrected for IL-6, SAA, CRP, d-dimer |
| Tenorio 2014 | Yes | At enrollment in ACTG: Age ≥13 yrs, HIV+, HIV-1 RNA load <400 copies/mL. Cases: death from a non-AIDS related event, MI, stroke, non-AIDS defining malignancy, serious bacterial infection. Controls matched on age, sex, pre-ART CD4 count, ART regimen | Yes | Yes | Number of potentially eligible patients not addressed | nd | Myocardial infarction according to ACTG definitions  Stroke | Adjusted for HIV RNA load, CD4 count, Hep B or C, CVD risk factors, injection drug use |
| Triant 2009 | Yes | Between 18 and 84 yrs with ≥1vist at Brigham and Women’s Hospital or Massachusetts General Hospital between 1997-2006, and CRP test ≤3 yrs, ≥1week before event | No | Yes | 70357 / 1648687  Inclusion based on available CRP test | nd | Acute myocardial infarction | Adjusted for demographic factors and CVD riks factors |
| **Study describing the same databases as one of the above mentioned studies, but with additional information** | | | | | | | | |
| Duprez 2012 (*additional to Nordell 2014)* | Yes | Patients with a CVD event from the SMART cohort. Age ≥13 yrs, CD4 count >350cells/m3. Matched 1:2 to controls based on age, sex, country, date of enrollment (≤3 months) | Yes | Yes | nd | Yes | CVD death, non-fatal MI (clinical and silent), non-fatal stroke, CHF, coronary revascularization, CAD requiring drug treatment, PAD | Corrected for age, gender, race, HIV-related factors, CV risk factors, ECG changes, hep B or C, Tx group. |
| **Carotid intima-media thickness** | | | | | | | | |
| Badiou 2008 | Yes | Consecutively included HIV+ patients out-/in patients clinic | Yes | Yes | 232/423 patients included. Based on available aliquots | nd | IMT of left FW CCA | Age, gender, tobacco consumption |
| Baker 2011 | Yes | HIV, naive to cART or solely cART, expected to survive >2yrs (SUN study). Inclusion if there was a baseline and 2-year CIMT measure | Yes | Yes | 270/659 participants at baseline had no 2 yr CIMT data available. | Yes | Difference between baseline and 2 yrs CIMT measurements  Far wall of the right distal common carotid artery | No multivariable analysis for hsCRP in relation to CIMT |
| Barbour 2013 | Yes | HIV infected, ≥40yrs, on stable ART ≥6monts | Yes | Yes | 125 of potential 158 patients analyzed (other no complete data) | nd | Right CCA IMT | Components of FRS, all potential markers (p <0,15 univariable) |
| Bonilla 2013 | Yes | LTNP: HIV≥5yrs, CD4>300cells/ml, no AIDS defining illness, never on ART | Yes | Yes | 13/16 patients had blood samples available. Other 3 not analyzed | Yes | Left and right CCA NW and FW, bulb, ICA. Mean values per segment: CCA/bulb/ICA | Pearsons correlation |
| Currier 2007 | Yes | From the A5078 prospective cohort: HIV, VL<10.000, all on ART, yes or no PI. Matched extensively, affected generalizability | Yes | Yes | 104/134 entered the extension at week 96 till week 144. 103/104 completed the final assessment at week 144 | nd | FW right CCA.  Progression of CIMT: yearly rate of change of at least 1 SD (≥0.0122mm/year) | Univariable logistic regression. Only univariable associations with a p-value ≤0.1 were included in multivariable analyis |
| Falcão 2012 | No | Consecutively HIV infected patients coming to an outpatient clinic | Yes | Yes | na  nd concerning potential eligible patients | nd | FW of left and right CCA.  Atherosclerosis if IMT>0,8mm | Univariable analysis.  Only variables with a p-value <0.25 were included in multivariable analysis |
| Freitas 2014 | Yes | HIV+ on stable ART visiting an outpatient clinic | Yes | Yes | na  nd concerning potential eligible patients | nd | Mean IMT of left and right CCA  Atherosclerosis if IMT>0.8mm | Unadjust analysis for CRP in relation to CIMT (correlation) |
| Hileman 2013 | Yes | HIV, >18yrs, not on ART, not expected to start ART during FU of 48wks (CD4 >400cells/mm3)  Excl: CVD, diabetes, active infection, pregnancy, breastfeeding | Yes | Yes | 15/85 HIV+ lost to FU (17.6%) | Yes | Mean-mean of left and right FW CCA at three angles and FW of bulb. | Multivariable analysis with all baseline factors with a p-value <0.15 |
| Hsue 2012 | No | Consecutive volunteers of the SCOPE cohort, enriched with ‘elite’ controllers | Yes | Yes | Yes 0-15%:  ICA13%, CCA 0%, bulb 4%, mean 15% | Yes | Average of NW and FW of left and right CCA, ICA and bulb  Plaque if IMT >1.5mm | Adjusted for demographics, CV risk factors and HIV-characteristics |
| Jeong 2011 | Yes | HIV, cART (>3 drugs) ≥6 mnts. Excl: obesity medication, CVD, malignancy, hypertension, infection, chronic liver or renal disease | Yes | Yes | na  nd concerning potential eligible patients | nd | Right and left CCA, bulb and ICA at 3 different points. Mean-IMT: average CCA left and right, max-IMT: greatest value of IMT.  Carotid plaque: focal wall thickening ≥50% of the surrounding vessel wall or ≥1.5mm. | Only univariable results |
| Joven 2006 | no | HIV+ from an outpatient clinic | Yes | Yes | na.  nd concerning potential eligible patients | Yes | Right and left CCA, bulb, ICA  Average CIMT value | Correlation |
| Kaplan 2012 | Yes | WIHS, and first report of HAART and available blood samples including 3 before and 3 after the use of HAART | Yes | Yes | 36% (46/127 had no CIMT available) | Yes | IMT of the FW of the right CCA | Adjusted for age, race, smoking, BMI, CD4 count, HIV RNA, class of ART |
| Longenecker 2014 | Yes | HIV infected, ≥18year, without DM or known CVD, on stable ART with VL <1000 copies/ml, increased T-cell activation or CRP >2mg/l, LDL ≤130mg/dl. First 60 subjects of the SATURN trial | Yes | Yes | na  nd concerning potential eligible patients | nd | Mean-mean and mean-max CCA-IMT.  Plaque: IMT>1.5mm or >50% thicker than the adjacent vessel  CCA-IMT >1.0 mm was defined as atherosclerosis | Correlation |
| Mangili 2014 | no | Patients from the CARE cohort. HIV infected patients without baseline diabetes, uncontrolled hypertension, myocardial infarction or stroke within the past 6 months | Yes | Yes | na  nd concerning potential eligible patients | nd | Mean of the maximum of near- and far wall CIMT were used for analysis: one for common carotid, one for internal carotid | Adjusted for age, race, FRS, cholesterol spectrum, BMI, homocysteine, Lp-PLA_2_, ApoE, NNRTI’s, waist circumference and blood pressure |
| Masia 2013 | Yes | Consecutive HIV infected patients, outpatient clinic, VL<200cp/ml, sexual transmitted | Yes | Yes | 136/157 patients. Based on availability of serological results for herpesviridae | Yes | Total CIMT: mean of CCA and bulb | Spearman correlation |
| Merlini 2012 | Yes | Consecutively enrolled HIV infected patients, on HAART (≥3 ARVs), >6mnths, VL<40 copies/ml at 2 consecutive assessments | Yes | Yes | 12/163 (7%) missing in multivariable analysis | nd | Mean value of the bifurcation, bulb, common carotid artery (left and right)  Normal IMT: ≤1mm, pathologic IMT >1mm. Divided in increased IMT and plaque | No confounder adjustment for the biomarkers in relation to CIMT |
| Piconi 2013 | no | Longitudinally enrolled, HIV infected man. Selected on FRS. Excl: use of statins | Yes | Yes | 76/79 cases analyzed. 4% ‘lost’ | Yes | Mean value of the distal CCA left and right. | Correlation |
| Portilla 2014 | Yes | HIV+ men, ≥18yrs, ART naive or ART and VL<50 copies in previous 6mnths, 2NRTI's+PI or NNRTI(-PI).  Excl: diabetes, chronic hepatitis C, active AIDS, drug use, psychiatric disorders | Yes | Yes | 89/109 potential eligible patients included. No loss to follow up | Yes  (CIMT measurements were done automatically) | Mean and max value of left and right CCA | Stepwise regression. All variable’s with p<0.05 in univariable analysis were included |
| Ross 2009 | Yes | HIV+, ≥21yrs, on stable ART≥24wks  Excl: known CVD, DM, opportunistic infection, cute or a chronic inflammatory condition | Yes | Yes | No  nd concerning potential eligible patients | Yes | Mean CCA and ICA left and right. | All variables if P <0.1 or if clinical significant were included in multivariable analysis |
| Ross 2014 | Yes | First 100 subjects who fulfilled the following criteria: HIV+, ≥18yrs, HIV<1000cp/ml, fasting LDL<130mg/dl, cumulative ART duration ≥6mnths,stable ART≥3mnths.  Excl: known CVD, statin use | Yes | Yes | No  nd concerning potential eligible patients | nd | Mean-mean CCA IMT  Plaque IMT >1.5mm | Linear regression: LpPLA1, age, male sex, current smoking, SBP, hsCRP |
| Sankatsing 2009 | Yes | Consecutive HIV patients visiting two outpatients clinics, on PI or NNRTI, stable >2yrs. VL<50cp/ml | Yes | Yes | nd concerning potential eligible patients | Yes | Right and left CCA, bulb, ICA  CIMT: average of the sum of the 3 right and left CCA FW | Covariates significantly associated with CIMT in univariable analysis were included in the multivariable analysis |
| Ssinabulya 2014 | Yes | HIV infected from 2 HIV clinics, ≥18years, CD4 >350 or on ART >7 years (41%). Exclusion: malignancy, infection, some drugs. No homogeneous | Yes | Yes | Yes: only 186/245 (76%) underwent CIMT measuring | nd | Overall CIMT for the CCA: mean values of 3 images at 3 different angles  Subclinical atherosclerosis: CIMT ≥0.78mm. | No. Bivariate analysis and correlation |
| Stein 2013 | Yes | Baseline evaluation of the AIDS Clinical Trials Group Study A5257. HIV infected, ≥18year, HIV RNA >1000, ART naive. Excl: known CVD, diabetes, uncontrolled hypertension, lipid lowering medication | Yes | Yes | No missing data Number of potential eligible patients not mentioned. | nd | CCA CIMT and bifurcation CIMT at the right sight.  Carotid artery lesion: >1.5mm wall thickness | Adjusted: candidate variable selection on the basis of Akaiki Information Criterion, clinical input and effect in univariable analysis |
| Tungsiripat 2011 | Yes | HIV+ with clinical lipoatrophy, stable ART>24months, HIV-Rna<5000.  Excl: pregnancy, diabetes, heart failure, cirrhosis, liver and kidney enzyme abnormalities | Yes | Yes | 9/71 (12.6%) lost to FU. Excluded from final analysis | Yes | Mean value of CCA and ICA. Plaque defined according to the prococol from the Cardiovascular Health Study | Correlation |
| Van Wijk 2006 | Yes | Men, 18-70yrs, HAART>12months, HIV RNA<10.000. Exclusion: opportunistic infection, malignancy, renal or liver disease, diabetes, lipid-lowering and antihypertensive agents  no homogeneous moment | Yes | Yes | na  nd concerning potential eligible patients | nd | Average of the left and right CCA | Spearman correlation |
| Westhorpe 2014 | Yes | HIV+, all on ART, VL <50cp/ml. Excl: PI <6mnths, use of statins, HIV RNA>50 <6mnths  No homogeneous moment | Yes | Yes | na  nd concerning potential eligible patients | Yes | Right and left CCA. The median value was used.  Subclinical atherosclerosis: median CIMT >0.7mm | Univariable. None of the markers significant, therefore not included in multivariable analysis. |
| **Studies describing the same databases as one of the above mentioned studies, but with additional information** | | | | | | | | |
| Hileman 2014 (additional to Hileman 2013) | Yes | HIV, >18yrs, not on ART, not expected to start ART during FU of 48wks (CD4 >400cells/mm3)  Excl: CVD, diabetes, active infection, pregnancy, breastfeeding | Yes | Yes | 42/85 analyzed. Other (50.6%) lost to follow up | Yes | Mean-mean of left and right FW CCA at three angles and FW of bulb. | Multivariable analysis with all baseline factors with a p-value <0.15 |
| Hsue 2006 (additional to Hsue 2012) | No | Patients from the SCOPE cohort, HIV+, on ART ≥1 yr or off ART ≥1 yr. Exclusion: acute infection, immune based therapy. 26.8% of HIV patients was involved in a previous study | Yes | Yes | No missing data Potential eligible part not addressed. | nd | Right and left both 6 predefined measurements. Plaque: IMT >1.5mm | Adjusted for traditional CV risk factors |
| Hsue 2009  (additional to Hsue 2012) | No | Patients from the SCOPE cohort, HIV+, no selection based on CVD risk. | Yes | Yes | No missing data Potential eligible part not addressed. | Yes | Average of NW and FW of left and right CCA, ICA and bulb | Traditional CV risk factors + additional factors associated with IMT in unadjusted analysis |
| Kelesidis 2012, 2013 | Yes | From the A5078 prospective cohort: HIV, VL<10.000, all on ART, whether or not on PI | Yes | Yes | 91/133 patients analyzed. FU till 96 weeks for 26% and FU of 144 wks for 74% | nd | Intima media thickness of the far wall of the distal right common carotid artery | Baseline covariates. Included in multivariable analysis if p<.05 |
| Kelesidis 2012, 2013 (additional to Currier 2007) | Yes | From the A5078 prospective cohort: HIV, VL<10.000, all on ART, Yes or no PI | Yes | Yes | 91/133 patients analyzed. FU till 96 weeks for 26% and FU of 144 wks for 74% | nd | IMT of FW of distal right CCA | Univariate analysis for markers in relation to CIMT |
| Parra 2010 (additional to Joven 2006) | Yes | Consecutive HIV-infected patients who came to the clinic, >18yrs. Excl: aids related infection, history of CVD | Yes | Yes | 152 of 187 patients analyzed. Exclusion not addressed. 19% ‘missing cases’ | nd | Median value of CCA, bulb, ICA. Subclinical atherosclerosis: IMT>0.8, plaque: IMT >1.5mm or protruding in the lumen >50% of surrounding CIMT value | Binary analysis. In multivariable analysis cardiovascular risk factors as well as HIV related factors were taken into account |
| AIDS: acquired immunodeficiency syndrome, ART: antiretroviral therapy, BMI: body mass index, CAD: coronary artery disease cART: combination antiretroviral treatment, CCA: common carotid artery, CHF: Congestive Heart Failure, CIMT: carotid intima-media thickness, CRP: C-reactive protein, CV: cardiovascular, CVD: cardiovascular disease, DM: diabetes mellitus, FRS: Framingham risk score, FU: follow up, FW: far wall, HAART: highly active retroviral therapy, HIV: humane immunodeficiency virus, ICA: internal carotid artery, IHD: Ischemic Heart Disease, IMT: intima-media thickness, LDL: low density lipoprotein, LTNP: Long term non-progressors, MI: myocardial infarction, na: not applicable, nd: no data, NRTI: nucleoside reverse transcriptase inhibitor, NNRTI: non-nucleoside reverse transcriptase inhibitor, NW: near wall, PAD: peripheral arterial disease, PI: protease inhibitor, SBP: systolic blood pressure, SD: standard deviation, Tx: treatment, VL: viral load | | | | | | | | |
